# *Clostridium butyricum* enhances colonization resistance against *Clostridioides difficile* by metabolic and immune modulation

**DOI:** 10.1038/s41598-021-94572-z

**Published:** 2021-07-22

**Authors:** Mao Hagihara, Tadashi Ariyoshi, Yasutoshi Kuroki, Shuhei Eguchi, Seiya Higashi, Takeshi Mori, Tsunemasa Nonogaki, Kenta Iwasaki, Makoto Yamashita, Nobuhiro Asai, Yusuke Koizumi, Kentaro Oka, Motomichi Takahashi, Yuka Yamagishi, Hiroshige Mikamo

**Affiliations:** 1 Department of Molecular Epidemiology and Biomedical Sciences, Aichi Medical University, Nagakute, 480-1195 Japan; 2 Department of Clinical Infectious Diseases, Aichi Medical University, Nagakute, 480-1195 Japan; 3 Miyarisan Pharmaceutical Co., Ltd., Saitama, 114-0016 Japan; 4 Department of Pharmacy, College of Pharmacy, Kinjo Gakuin University, Nagoya, 463-8521 Japan; 5 Departments of Kidney Disease and Transplant Immunology, Aichi Medical University, Nagakute, 480-1195 Japan

**Keywords:** Immunology, Microbiology, Gastroenterology

## Abstract

*Clostridioides difficile* infection (CDI) represents the leading cause of nosocomial diarrhea worldwide and is associated with gut dysbiosis and intestinal damage. *Clostridium butyricum* MIYAIRI 588 (CBM 588) contributes significantly to reduce epithelial damage. However, the impacts of CBM 588 on antibacterial therapy for CDI are not clear. Here we show that CBM 588 enhanced the antibacterial activity of fidaxomicin against *C. difficile* and negatively modulated gut succinate levels to prevent *C. difficile* proliferation and downregulate tumor necrosis factor-α (TNF-α) producing macrophages in the colon lumina propria (cLP), resulting in a significant decrease in colon epithelial damage. Additionally, CBM 588 upregulated T cell-dependent pathogen specific immunoglobulin A (IgA) via interleukin (IL)-17A producing CD4^+^ cells and plasma B cells in the cLP, and Th17 cells in the cLP enhanced the gut epithelial barrier function. IL-17A and succinic acid modulations with CBM 588 enhance gut colonization resistance to *C. difficile* and protect the colon tissue from CDI.

## Introduction

*Clostridioides difficile* is an infectious, gram-positive, spore-forming, *Bacillus* microorganism. This pathogen is present in the gastrointestinal tract and causes symptoms ranging from mild diarrhea to pseudomembranous colitis, toxic megacolon, and death due to toxin production^1,2^. *C. difficile* infection (CDI) causes severe, potentially life-threatening intestinal inflammation, especially in hospitalized patients^3^. High densities of bacterial species colonize the gut and constitute the microbiome^4^. To avoid CDI, a healthy gut microbiome modulates its metabolites to interfere with *C. difficile* growth^5,6^. In addition to metabolic functions, the gut microbiome can activate the host immunity, indirectly preventing the colonization and growth of many enteric pathogens^7^. These associations play an important role in protecting the host’s mucosal immune function, epithelial barrier integrity, gastrointestinal motility, and nutrient absorption^8^. However, the precise mechanism by which members of a healthy microbiome compete *with C. difficile* is not fully understood.

*Clostridium butyricum* is a gram-positive obligate anaerobic *Bacillus* inhabiting soil, as well as animal and human intestines. Specifically, *C. butyricum* MIYAIRI 588 (CBM 588) has been used to treat various human gastrointestinal diseases in Japan^9^. CBM 588 administration reduced antibiotic-induced gut epithelial damage. Additionally, in a previous *in vivo study*, CBM 588 reduced superficial epithelial necrosis and the presence of inflammatory cells^10^. In the colonic lamina propria (cLP), CBM 588 induced interleukin (IL)-17A-producing γδ T cells and IL-17A-producing CD4^+^ cells^11^. However, the efficacy of these protective mechanisms in the treatment of CDI remains unknown.

To elucidate the impact of CBM 588 on treatment of CDI, we investigated the immunological and metabolic interactions between the host and gut microbiome during CDI. This study revealed two novel gut microbiome and colon protective mechanisms of CBM 588 treatment. First, CBM 588 modulation of the gut microbiome resulted in a reduction of gut succinate level, along with suppression *of C. difficile* proliferation and colon inflammation. Second, CBM 588 enhanced T cell-dependent B cell activation to enhance pathogen-specific immunoglobulin (Ig) A production by upregulating IL-17A-producing CD4^+^ cells in the cLP. At the same time, Th17 cells in the cLP enhanced the gut epithelial barrier function. Therefore, this study provides new insights into the prevention and treatment of CDI with CBM 588 through enhanced colonization resistance against *C. difficile*.

## Results

### *C. butyricum *enhanced *C. difficile* colonization resistance in the gut and attenuated gut inflammation when used with an anti-*C. difficile* antibiotic agent

**Figure 1:**
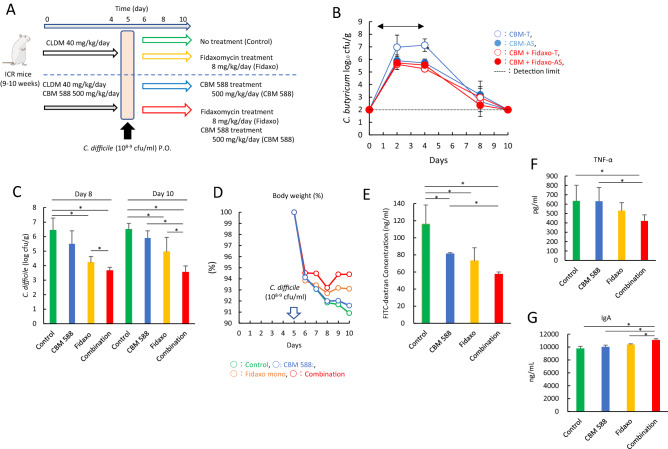
*C. butyricum* enhanced *C. difficile* colonization resistance in the gut and attenuated gut inflammation when used with an anti-*C. difficile* antibiotic agent. (**A**) Experimental design of the *Clostridioides difficile* infection model with 9- to 10-week-old ICR Swiss mice. (**B**) Enumerating *C. butyricum* in feces. (open circle): total colony count in CBM 588 administration group (CBM 588-T), (filled circle): spore colony count in CBM 588 monotherapy group (CBM 588-AS), (open circle): total colony count in combination group (Combination-T), (filled circle): spore colony count in combination group (Combination-AS). The testing detectable level is above 2.0 (log amount) per gram of feces, data shown as mean ± SD (n = 5–10 per group). (**C**) Enumerating *C. difficile* in feces at day 8 and 10. 1: control group (Control), 2: CBM 588 monotherapy group (CBM 588), 3: fidaxomicin monotherapy group (Fidaxo), and 4: combination (CBM 588 + fidaxomicin) group (Combination). The testing detectable level is above 2.0 (log amount) per gram of feces, data shown as mean ± SD (n = 5–10 per group). (**D**) Weight changes of control group (green), CBM 588 administration group (blue), fidaxomicin administration group (yellow), and combination group (red) over the duration of the study (from *C. difficile* inoculation to day 10), data shown as mean (n = 5 per group). (**E**) Intestinal permeability was determined. All values are mean ± SD (n = 5). (**F**) Tumor necrosis factor-α (TNF-α) and (**G**) Immunoglobulin A (IgA) protein concentrations in mice colon tissue detected at day 8. All values are mean ± SD (n = 5–10).

To determine whether colonization by CBM 588 has an immunomodulatory and metabolic role in regulating gut homeostasis during CDI, we administered fidaxomicin, a frequently used narrow-spectrum first-in-class macrolide antibacterial drug indicated for the treatment of CDI in adults, and/or CBM 588 to ICR mice for 4 days (Fig. 1A). Colonization of *C. butyricum* was retained in the colon, even after fidaxomicin administration (Fig. 1B). The spore colony counts for the CBM 588 and combination (fidaxomicin + CBM 588) groups were comparable throughout the study period. Notably, the combination therapy group showed significantly lower *C. difficile* colony counts in fecal samples at days 8 and 10, compared with the fidaxomicin monotherapy group (*p* < 0.05) (Fig. 1C). Moreover, during the study period, weight loss in ICR mice was attenuated with CBM 588 administration (Fig. 1D). The combination therapy group had the lowest gut permeability (Fig. 1E) and colon pathology score (Fig. S-1A). CBM 588 modulated cytokine expression, thereby regulating gut inflammation. During CDI, expression of pro-inflammatory cytokines, such as tumor necrosis factor-α (TNF-α), was lower in the combination therapy group than in the fidaxomicin monotherapy group (Fig. 1F), while the expression of IL-10, an anti-inflammatory cytokine, was higher in the CBM 588 monotherapy and combination therapy groups (Fig. S-1B). Additionally, the combination therapy group showed the highest IgA production in the colon (Fig. 1G).

### *C. butyricum* modulated the gut microbiome composition under fidaxomicin treatment and increased *Lactobacillus* spp. and *Lactococcus* spp.

**Figure 2:**
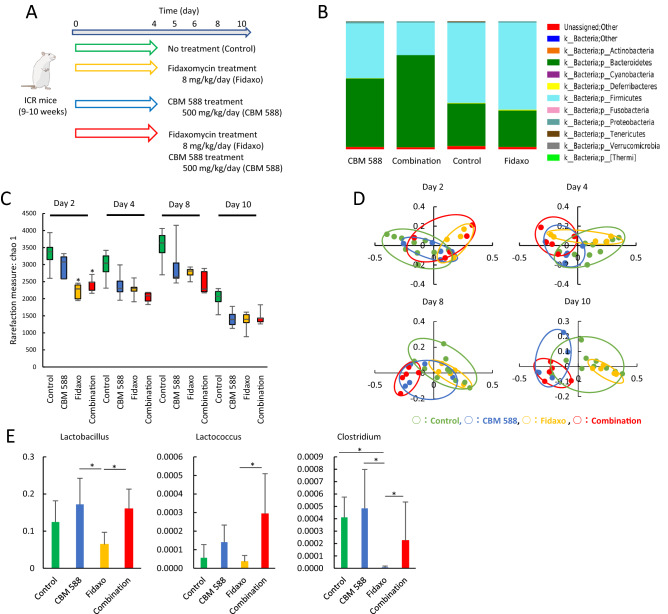
*C. butyricum* modulated the gut microbiome composition under fidaxomicin treatment and increased *Lactobacillus* spp. and *Lactococcus* spp. (**A**) Experimental design for gut microbiome analysis with 9- to 10-week-old ICR Swiss mice. (**B**) Bacterial compositions in different experimental groups at the phylum level. All values are mean (n = 5). (**C**) Comparison of the Shannon index of different groups. The box and whiskers represent the smallest and largest values, with the median in the center of each box (n = 5). (**D**) Principal Coordinate Analysis (PCoA) based on weighted Unifrac distances among CBM 588 monotherapy group (CBM 588), fidaxomicin monotherapy group (Fidaxo), and combination group (Combination). (**E**) Effect of fidaxomicin and/or CBM 588 administration on relative species abundance (≥ 0.001%) in the fecal samples. After quality filtering steps, three species (*Lactobacillus* spp., *Lactococcus* spp., and *Clostridium* spp.) were considered to have significantly higher relative abundance (%) in the combination group, compared with the fidaxomicin administration group at day 8. Data represent the mean values of relative abundances ± SD (n = 5–10).

To reveal the impact of CBM 588 on the gut microbiome during treatment of CDI, we administered CBM 588 and fidaxomicin to non-infected mice (Fig. 2A). Changes in the murine gut microbiome at the phylum level, after 4 days, are shown in Fig. 2B. Bar graphs depict the mean percentage abundance of bacterial families (> 1% relative abundance). The intestinal flora mainly consisted of members of the *Firmicutes* and *Bacteroidetes* phyla in all groups. Fidaxomicin administration did not change the gut microbiome at the phylum level. However, the biggest change for the non-infected mice was observed in the combination therapy group. The combination treatment resulted in an increase in the *Bacteroidetes* phylum (*p* < 0.05), relative to the control group, whereas the abundance of members of the *Firmicutes* phylum decreased (*p* < 0.05).

To determine bacterial richness and diversity in the gut microbiome, we calculated α-diversity and β-diversity based on fecal samples from all groups, and found that fidaxomicin exposure significantly decreased α-diversity at day 2 (*p* < 0.05) (Fig. 2C). A decrease in bacterial α-diversity was observed in both the fidaxomicin monotherapy and combination therapy groups and did not recover during the study period. Principal coordinate analysis (PCoA) based on the weighted UniFrac distance metric is shown in Fig. 2D. There was no significant change in the bacterial community composition between the CBM 588 administration and control groups on days 2, 4, 8, and 10. However, bacterial communities in the fidaxomicin-treated groups clustered separately from the combination therapy group on days 8 and 10 (*p* < 0.05), whereas bacterial communities in fecal samples of the CBM 588 and combination therapy groups did not cluster separately from each other during the study period (Fig. 2D). We then investigated murine gut microbiome changes at the genus and species levels on day 4 (Fig. S-2A). CBM 588 administration resulted in increase in *Lactobacillus* and *Lactococcus* spp., while *Clostridium* spp. were reduced in the fidaxomicin-treated group (*p* < 0.05) (Fig. 2E). The relative abundance (%) of three genera (*Lactobacillus*, *Lactococcus,* and *Clostridium*) in the combination therapy group was significantly higher than that in the fidaxomicin-treated group (Fig. 2E).

### C. butyricum changed gut microbiome profiles to modulate gut succinate level

**Figure 3:**
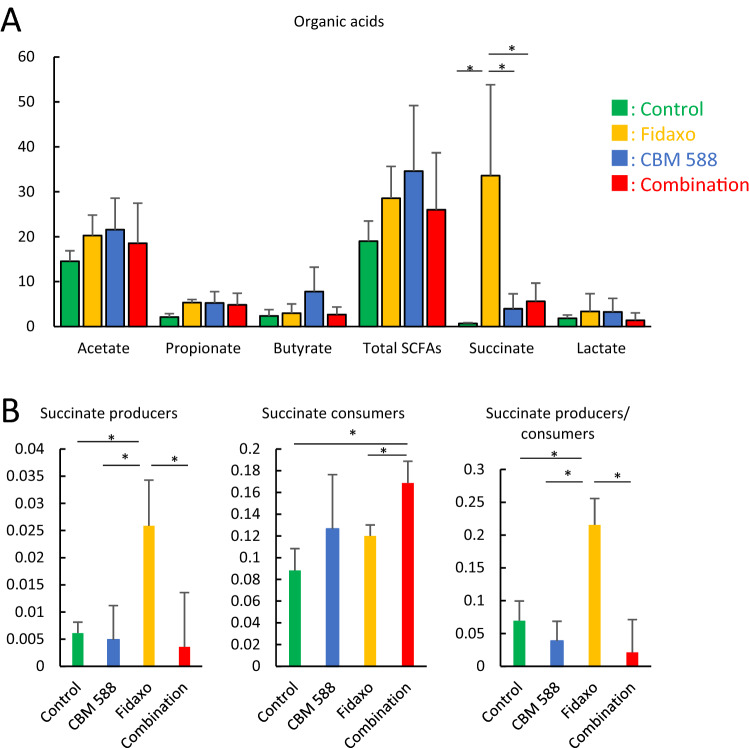
*C. butyricum* changed gut microbiome profiles to modulate gut succinate level. (**A**) Concentration of organic acids in fecal samples taken at day 4. (green: control group, yellow: fidaxomicin group, blue: CBM 588 group, red: combination group) All values are mean ± SD (n = 5–10). (**B**) Effect of fidaxomicin and CBM 588 administration on relative succinate producer and consumer species abundance in the fecal samples. Data represent the relative abundance (%) mean values of relative abundances ± SD (n = 5–10). Succinate producer: *Propionibacterium* spp., *Alistipes* spp., *Paraprevotella* spp., *Parabacteroides* spp., *Blautia* spp., *Faecalibacterium* spp., *Citrobacter* spp., *Succinivibrio* spp., *Akkermansia* spp., Succinate consumer: *Bacterides* spp., *Clostridium* spp., *Phascolarctobacterium* spp., *Ruminococcus* spp., *Dialister* spp., *Veillonella* spp. (reference 12).

As microbiome-derived metabolites act both as nutrients and as messenger molecules to the host, we focused on metabolic alterations in the resident microbiome to elucidate how CBM 588’s modulation of the gut microbiota impacted metabolic functions during CDI. The organic acids in the feces at day 4 are shown in Fig. 3A. The fidaxomicin monotherapy group showed the highest succinate concentration, whereas succinate concentration was decreased in the combination therapy group (*p* < 0.05). Fernández-Veledo et al. suggested that succinate, a gut microbiome-derived metabolite, plays a key role in governing intestinal homeostasis and energy metabolism. Some gut microbiome species are succinate producers and consumers^12^. Hence, we extracted succinate producer and consumer species from the same gut microbiome data, and found that fidaxomicin promoted succinate production (*p* < 0.05) and reduced succinate producers (Fig. 3B). However, CBM 588 treatment reduced gut succinate to normal levels and modulated succinate producer/consumer levels. These results support the hypothesis that CBM 588 inhibits increase in gut succinate levels via gut microbiome modulation.

### High gut succinate levels induce C. difficile proliferation and cause gut inflammation

**Figure 4:**
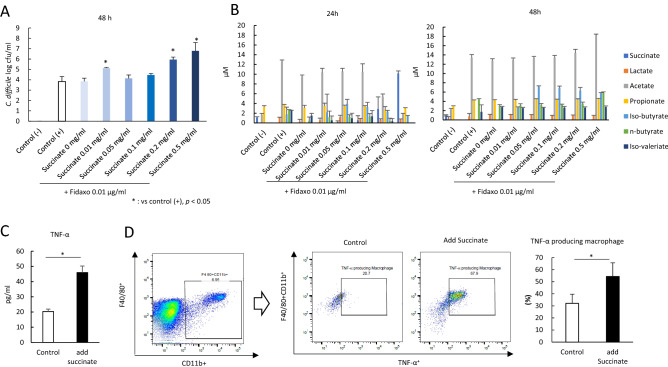
High gut succinate levels induce *C. difficile* proliferation and cause gut inflammation. (**A**) Assessment of *C. difficile *in vitro study at 48 h. 1: control group without *C. difficile* inoculation (Control (−)), 2: control group with *C. difficile* inoculation (Control (+)), 2–8: treatment group with succinate 0–0.5 mg/ml under fidaxomicin 0.01 μg/ml. The testing detectable level is above 2.0 (log amount) per ml of medium, data shown as mean ± SD (n = 5 per group). (**B**) Concentration of organic acids in the medium. Samples were taken at 24 h (left) and 48 h (right). All values are mean ± SD (n = 5). (**C**) Tumor necrosis factor-α (TNF-α) protein concentration in mice colon tissue detected at day 5. All values are mean ± SD (n = 5–10). (**D**) Representative flow cytometry plots of identified macrophages (F40/80^+^/CD11b^+^), and tumor necrosis factor-α (TNF-α) producing macrophage (F40/80^+^/CD11b^+^/TNF-α^+^) expression among lymphocytes in the colonic lamina propria (cLP) in the control group (Control), and the other group with succinate administration for 4 days (Add succinate). Percentage of TNF-α secreting macrophages in macrophages (right).

To reveal the impact of succinate concentration on *C. difficile* proliferation, we conducted an in vitro study. We found that *C. difficile* proliferation was enhanced by the addition of succinate to the medium (Fig. 4A). Simultaneously, analysis of organic acids in the medium after 24 h and 48 h incubation of cultures revealed that *C. difficile* depleted succinate from the medium (Fig. 4B). Next, we conducted an in vivo experiment to evaluate the impact of succinate administration on colon tissue. We supplemented the drinking water of the mouse with 150 mM succinate for 4 days. Subsequently, the succinate level in fecal samples increased to 40 mM on day 4, succinate administration induced weight loss (Fig. S-3A), and there was upregulation of pro-inflammatory cytokine TNF-α expression in the cLP (Fig. 4C), while the expression of IL-4, IL-6, IL-10, and transforming growth factor (TGF)-β_1_ decreased (Fig. S-3B). Remarkably, TNF-α-producing macrophages (F40/80^+^/CD11b^+^/TNF-α^+^) in the cLP increased with succinate administration (*p* < 0.05) (Fig. 4D). TNF-α has been shown to play a pivotal role as a master regulator of inflammatory cytokine production^13^. Therefore, our results suggest that microbiome-induced high succinate levels cause gut inflammation.

### *C. butyricum* upregulated IL-17A-producing CD4^+^ cells in the cLP to increase IgA production during CDI

**Figure 5:**
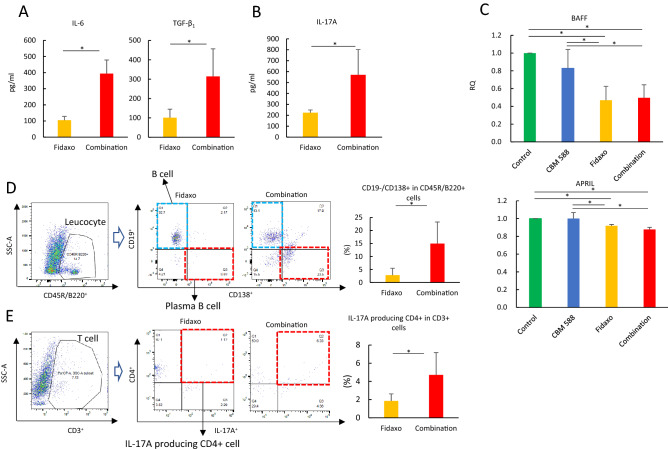
IL-17A plays important role to enhance antibacterial activity of combination therapy with *C. butyricum* against *C. difficile* and activate plasma B cell to enhance IgA production. (**A**) Interleukin (IL)-6, Transforming growth factor-β_1_ (TGF-β_1_) and (**B**) IL-17A protein expressions in mice colon lumina propria (cLP) detected at day 8. All values are mean ± SD (n = 5–10). (**C**) The relative RNA expression of genes encoding proliferation-inducing ligand (APRIL) and B-cell activating factor (BAFF) in colon tissues of mice, detected by qPCR. All values are mean ± SD (n = 5–10). (**D**) Representative flow cytometry plots of B cell (CD45R/B220^+^/CD19^+^/CD138^-^) and plasma B cell (CD45R/B220^+^/CD19^−^/CD138^+^) expressions among leukocytes in the colonic lamina propria (cLP) in the fidaxomicin monotherapy group (Fidaxo), and combination group (Combination). Percentage of plasma B cell among leukocytes in cLP (right). (**E**) Representative flow cytometry plots of IL-17A producing CD4^+^ cell (CD3^+^/CD4^+^/IL-17A^+^) expression among T cells in the cLP in the fidaxomicin monotherapy group (Fidaxo), and combination group (Combination). Percentage of IL-17A producing CD4^+^ cells among CD4^+^ cells among T cells in cLP (right).

In our study, TGF-β_1_ and IL-6, which can enhance the differentiation of Th17 cells from naïve T cells^14^, were upregulated in the combination therapy group after CBM 588 administration (Figs. 5A and S-4). Consequently, in the combination therapy group we observed upregulation of IL-17A in the cLP (Fig. 5B). Additionally, in our in vivo study using the murine CDI model, fidaxomicin exposure resulted in the downregulation of RNA expression of proliferation-inducing ligand (APRIL) and B-cell activating factor (BAFF), and fidaxomicin monotherapy and combination therapy groups showed similar RNA expression levels at day 8 (Fig. 5C). In Peyer's patches (PPs), Th17 cells induced the development of germinal center B cells, which produced antigen-specific high-affinity T cell-dependent IgA from T follicular helper (Tfh) cells^14,15^. In our study, the combination therapy group showed upregulation of plasma B cells (CD45R/B220^+^/CD19^−^/CD138^+^) in the cLP (*p* < 0.05) (Fig. 5D). Additionally, CBM 588 administration resulted in the upregulation of IL-17A-producing CD4^+^ cells (CD3^+^/CD4^+^/IL-17A^+^), which was the major source of gut-protective IL-17A in the cLP for the combination therapy group (*p* < 0.05) (Fig. 5E).

**Figure 6:**
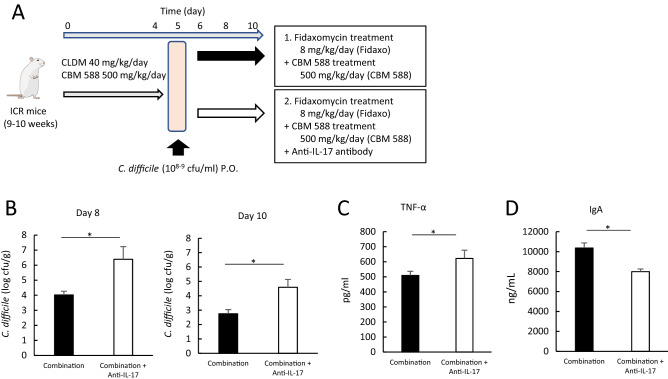
*C. butyricum* upregulated IL-17A in the cLP to increase IgA production under CDI. (**A**) Experimental design of fidaxomicin and CBM 588 administration with/without anti-IL-17A antibody in 9- to 10-week-old ICR Swiss mice. (**B**) Assessment of *C. difficile* in feces at day 8 and 10. 1: combination (CBM 588 + fidaxomicin) group (Combination), 2: combination group + anti-IL-17A antibody. The testing detectable level is above 2.0 (log amount) per gram of feces, data shown as mean ± SD (n = 5–10 per group). (**C**) Tumor necrosis factor-α (TNF-α) and (**D**) Immunoglobrin A (IgA) protein concentrations in mice colon tissue detected at day 8. All values are mean ± SD (n = 5–10).

Furthermore, to reveal the role of Th17 signaling in combination therapy, we added an anti-IL-17A antibody to the combination therapy group (Fig. 6A). Administration of the anti-IL-17A antibody attenuated the antibacterial activity of the combination therapy against *C. difficile* (Fig. 6B)*.* Additionally, administration of the anti-IL-17A antibody enhanced weight loss due to CDI (Fig. S-5A) and induced the upregulation of colon pro-inflammatory markers, such as TNF-α, while IL-10 was decreased (Figs. 6C and S-5B). Moreover, we observed a reduction in IgA production in the colon tissue (Fig. 6D). Collectively, our results suggest that CBM 588 enhances anti-CDI effects by upregulating pathogen-specific IgA in plasma B cells. Th17 cells play an important role in enhancing the antibacterial activity of the combination therapy.

### Th17 cells activated with CBM 588 reinforced colon epithelial cells during CDI

**Figure 7:**
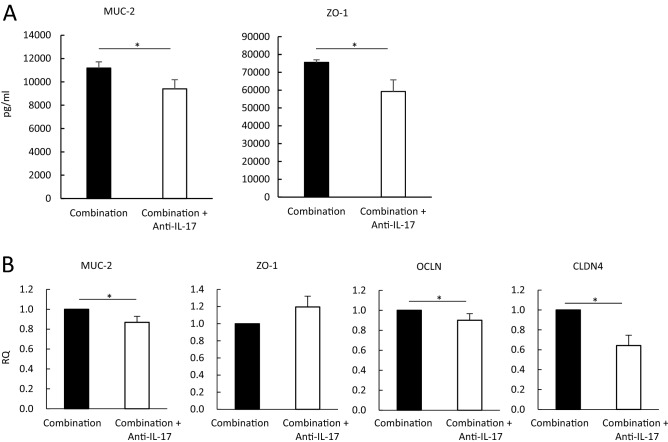
Th17 cells activated with *C. butyricum* strengthen colon epithelial cells under CDI. (**A**) The mucin-2 (MUC-2) and zonula occludens (ZO-1) protein concentrations in each animal group were detected. 1: combination (CBM 588 + fidaxomicin) group (Combination), 2: combination group + anti- IL-17A antibody. (**B**) The relative RNA expression of genes encoding MUC-2, claudin (CLDN4), ZO-1, and occludin (OCLN) in mice colon tissue, detected by qPCR. All values are mean ± SD (n = 5–10).

To further characterize the protective effects of IL-17A induced by CBM 588 on the epithelial layer during antibiotic administration in mice, we determined mucin (MUC)-2, zonula occludens-1 (ZO-1), claudins-4 (CLDN4), and occludin (OCLD) expression levels in the colon tissue of mice treated with the combination (fidaxomicin + CBM 588) therapy with or without anti-IL-17A antibody. Our results indicate that anti-IL-17A antibody administration downregulated MUC-2 and ZO-1 protein expression under the combination therapy (Fig. 7A). Anti-IL-17A antibody administration also downregulated the RNA expression levels of MUC-2, OCLN, and CLDN (Fig. 7B). ZO-1 gene data appeared to be inconsistent with ZO-1 protein expression. However, the ZO-1 gene expression data were not significantly different. Several studies have revealed impaired tight junction (TJ) complexity and downregulation of TJ proteins in ulcerative colitis (UC), which could contribute to the dysfunction of the intestinal barrier. In this study, CBM 588 increased IL-17A expression in the cLP and the upregulation of mucin production and TJ protein expression levels, which have been found to lead to greater epithelial barrier integrity^16,17^.

## Discussion

The gut immune system and microbiome are both critical components of the intestinal barrier function in the prevention of CDI^18^. CBM 588 has a major impact on the treatment of gastrointestinal diseases^9^ and protects against epithelial damage caused by antibiotics^11^. However, the role of host metabolite- and immunity-mediated colonization resistance to *C. difficile*, conferred by the gut microbiome due to CBM 588 administration, remains overlooked.

Our gut microbiome analysis revealed that fidaxomicin had a minor impact on the gut microbiome, as previously shown^19^. However, CBM 588 combination therapy with fidaxomicin revealed that CBM 588 has some effects on the gut microbiome composition. As a previous study revealed, CBM 588 increased *Lactobacillus* and *Lactococcus* spp. (Fig. 2A)^10^. *Lactobacillus* spp. have gut immune modulatory effects that attenuate gut inflammation, enhance antigen-specific IgA secretion, and induce Tfh cells in mice^20^. Additionally, *Lactobacillus* exhibits a potent protective effect on the intestinal barrier^21^. Hence, our current microbiome study suggests that CBM 588 administration can have a positive impact on the gut colonization resistance to *C. difficile*.

In addition, like other antibiotics, fidaxomicin treatment increased gut succinate levels, which enhanced *C. difficile* expansion and gut inflammation (Fig. 4A,C). However, CBM 588 administration resulted in a reduction of gut succinate levels to normal (Fig. 3A), and several studies have revealed a clear association between gut microbiome disturbances, such as antibiotic-induced dysbiosis, and succinate accumulation in the gut lumen^22,23^. Fidaxomicin has a specific antibiotic activity, especially against Clostridia, which is thought to be advantageous for the use of this antibiotic since it has a slight impact on the gut microbiome^24^. However, our study suggested that fidaxomicin disturbed the gut succinate producer/consumer balance mainly because of its specific antibiotic activity against Clostridia, one of the major succinate consumers in the gut microbiome (Fig. 3B)^25^. Moreover, reduction of luminal succinate levels resulted in restored colonization resistance against *C. difficile*^26^. Taken together, fidaxomicin did not affect CBM 588 colonization in the gut, but disturbed the gut succinate balance. However, administration of CBM 588, an isolate that belongs to the *Clostridium* spp., prevented the gut microbiome from altering the gut succinate level, even under fidaxomicin administration. In addition to higher succinate concentration in the gut, *C. difficile* proliferation on epithelial cells induced inflammation and leaky gut^18^. Hence, inflammation markers, such as TNF-α, reduced depending on the *C. difficile* density in the gut. CBM 588 has relatively low antibacterial activity against *C. difficile*^27^, while having protective effects on epithelial cells of the gut. Hence, we propose that the combination therapy showed synergistic effects in reducing gut inflammation, resulting in anti-inflammatory effects and enhanced colonization resistance to *C. difficile*. In both mice and humans, inflammatory bowel disease (IBD) causes an increase in fecal succinate levels, which has been linked to disease activity^12,28^; however, the cause or effect remains unresolved. Hence, CBM 588 administration-linked modulation of gut succinate levels and gut microbiome can be an effective treatment for diseases related to gut dysbiosis, including CDI.

Furthermore, CBM 588 administration enhanced the antibiotic activity of fidaxomicin against *C. difficile* (Fig. 1C). Antigen-specific IgA through the secretion of TGF-β_1_ plays a major role in the host defense against infections in the gut mucosal tissue^29^. Previous studies have revealed that dendritic cells (DCs) in PPs induce antigen-non-specific IgA class-switch recombination (CSR) through a T cell-independent pathway by producing retinoic acid, APRIL, and BAFF^30^. In contrast, antigen-specific IgA is induced by helper T cells activated by DCs through the secretion of TGF-β_1_, IL-4, and the CD40 ligand^28^. In our in vivo study with the murine CDI model, fidaxomicin exposure resulted in the downregulation of RNA expression of APRIL and BAFF; the fidaxomicin monotherapy and combination therapy groups showed similar RNA expression levels on day 8 (Fig. 5C). Hence, as several studies with other probiotics have demonstrated^20^, orally administered CBM 588 enhanced T cell-dependent antigen-specific IgA production in the colon (Figs. 1G, 6D). CBM 588 increased the proportion of plasma B cells (Fig. 5D) and IL-17A-producing CD4^+^ cells in the cLP (Fig. 5E). Furthermore, CBM 588 increased the protein expression of TGF-β_1_, IL-17A, and IL-6 associated with Tfh cell differentiation into plasma B cells (Fig. 5A and 5B). These results suggest that Th17 cells are induced by CBM 588 administration and play an important role in enhancing the resistance to *C. difficile* colonization by combination therapy. Orally administered CBM 588 enhanced antigen-specific IgA production in the cLP.

Th17 cells contribute to the maintenance of host gut immune homeostasis, at least partially, via IL-17A-induced expression of epithelial polymeric immunoglobulin receptors (pIgR). This results in increased IgA secretion into the lumen^31^. Furthermore, the current in vivo study showed that CBM 588 administration upregulated TJ proteins and MUC-2-producing proteins in the CDI model (Figs. 7A and 7B). Collectively, our results suggest that CBM 588 administration stimulates IL-17A expression in the cLP. This led to the induction of epithelial pIgR expression, which increased IgA secretion into the lumen. Moreover, stimulated IL-17A expression in the cLP enhanced gut epithelial protection by strengthening TJs.

Our study revealed some novel mechanistic insights into how CBM 588 modulates anti-inflammatory and epithelial protective effects against CDI. Nevertheless, this study had some limitations. First, the gut microbiome of mice is not identical to the human gut microbiome^32^. Hence, further research is needed to investigate whether these results can be reproduced in humans. Second, intra-day variation was larger than expected in our gut microbiome study, especially on day 10 (Fig. 2C). The α-index values between the control group on days 2 and 10 showed a significant difference (*p* < 0.05), while these data showed a similar tendency. These facts might reflect that the intra-day differences in our gut microbiome data were larger than the variation observed between the groups. Third, the precise reason why *C. butyricum* was not inhibited by fidaxomicin was unclear. However, in our in vivo study we observed live *C. butyricum* isolates in fecal samples, even under fidaxomicin treatments (8 mg/kg/day) (Fig. 1B). We expected that fidaxomicin would have antibacterial activity against *C. butyricum*, but the distribution of the agent was uneven in the gut. Hence, *C. butyricum* could inhabit spaces with low to zero concentration of fidaxomicin.

## Conclusions

**Figure 8:**
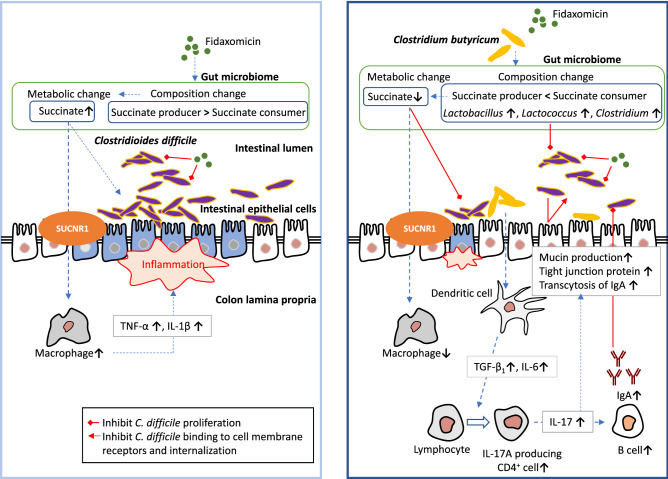
Proposed mechanisms to show colonization resistance induced by *Clostridium butyricum* MIYAIRI 588 strain (CBM 588) against *Clostridioides difficile* infection. Not only CBM 588 modulated gut microbiome, CBM 588 negatively modulated gut succinate levels to prevent *C. difficile* proliferation and downregulate tumor necrosis factor-α (TNF-α) producing macrophages in the colon lumina propria (cLP), resulting in a significant decrease in colon epithelial damage. Additionally, CBM 588 upregulated T cell-dependent pathogen specific immunoglobulin A (IgA) via interleukin (IL)-17A producing CD4^+^ cells and plasma B cells in the cLP, and Th17 cells in the cLP enhanced the gut epithelial barrier function (as shown to the right). SUCNR1: succinate receptor 1.

In conclusion, our study revealed the effectiveness of CBM 588 in the treatment of CDI (Fig. 8). CBM 588 protects against *C. difficile* expansion in the gut by regulating the gut microbiome and metabolic function. CBM 588 also decreased gut antibiotic-induced succinate levels; a novel function. In addition, CBM 588 modulates cytokines to protect colon epithelial cells from inflammation. Finally, CBM 588 upregulated IL-17-producing CD4^+^ T cells, which activated plasma B cells, leading to enhanced pathogen-specific IgA production and enhanced gut epithelial protection with strengthened TJs. These mechanisms lead to the enhancement of *C. difficile* colonization resistance in the gut, and these results provide new insights into the prevention and treatment of CDI.

## Methods

The study was reviewed and approved by the ethics committee of the Aichi Medical University (#2019–108). Also, this in vivo study was carried out in compliance with the ARRIVE guidelines 2.0 (https://arriveguidelines.org). Animal experimental procedures including euthanasia methods were performed according to The Japanese College of Laboratory Animal Medicine (JCLAM)’s publication guide for care and use of laboratory animals (https://www.jalam.jp/) and American Veterinary Medical Association (AVMA) Guidelines for the Euthanasia of Animals (2020)^33^.

### Medicine

CBM 588 bacterial powder was used for all in vivo studies at a concentration of 2.2*10^10^ cfu/g (Lot 61GT: MIYARISAN Pharmaceutical Co. Ltd., Tokyo, Japan). Clindamycin for injection was purchased from Pfizer Japan Inc (Tokyo, Japan). Analytical grade fidaxomicin was purchased from Selleck Chemicals Inc (Houston, TX, USA). and diluted with dimethyl sulfoxide (FUJIFILM Wako Chemical Co. Ltd., Osaka, Japan) with 500 mg/mL used as the stock solution. Immediately before each in vivo experiment, CBM 588 powder was weighed and reconstituted with sterile water. Clindamycin and fidaxomicin were further diluted to achieve the desired concentration with distilled water. Fidaxomicin solution was stored 4ºC and discarded 12 h after reconstitution. Neutralizing antibody to IL-17A was generated at BD Biosciences (Franklin Lakes, NJ, USA).

### Microorganism

This study used *Clostridioides difficile* strain ATCC43255 for all in vivo and in vitro study.

### Animals and housing

Pathogen-free, female ICR Swiss mice (9–10 weeks) weighing approximately 30 g were obtained from Charles River Laboratories Japan, Inc. (Yokohama, Japan) (Total N = 90). Mice were maintained and utilized under the National Research Council recommendations and were provided food and water ad libitum^10^.

### Method of administration

Twenty female ICR Swiss mice were divided into four groups (n = 5): (i) control group, (ii) CBM 588 single administration group, (iii) fidaxomicin single administration group, and (iv) combination group (CBM 588 + fidaxomicin). Before *C. difficile* administration, only clindamycin was administered to groups i and iii, and clindamycin + CBM 588 were administered to groups ii and iv for 4 days. CBM 588 was administered by oral gavage at 500 mg/kg/day (3.4 × 10^8^ cfu/mice/day). Clindamycin was given to mice via a sonde at 40 mg/kg/day^10^. Fidaxomicin was given to mice via a sonde at 8 mg/kg/day. CBM 588 powder was dissolved into sterilized water, and the suspension was mixed and given to mice via a sonde. For combination groups, CBM 588 and antibiotics were dissolved into sterilized water separately. Mice were given the suspension twice a day, at 10 a.m. and 4 p.m., using a half dose each time, over the course of 4 days. Anti-Mouse IL-17A (clone TC11-18H10) was dosed at 500 μg i.p. once at Day 0^11^.

### Assessment of physiological condition

Weight loss and stool consistency were assessed daily to determine any possible physical changes. Body weight was recorded every other day. These data were reported as percentage of weight loss from initial body weight^10^.

### Histological examination

Histological examination was performed as previously described^10^. Briefly, on day 8, subsets of mice from each group were sacrificed to check for histological changes in the colon tissue. At harvest, the mice were euthanized by an overdose of CO_2_ followed by cervical dislocation. Fixed sections of colonic tissues were embedded in paraffin. These tissues were then cut into 3-μm sections and stained with hematoxylin and eosin for histological analysis via light microscopy. The samples were blindly evaluated by a skilled pathologist, focusing on the following parameters associated with colitis: (1) neutrophil margination and tissue infiltration, (2) hemorrhagic congestion and edema of the mucosa, and (3) epithelial cell damage. A score of 0–3, denoting increasingly severe abnormality, was assigned to each of these parameters and their combined scores were graphed^34^.

### Analysis of intestinal permeability in mice

To determine in vivo intestinal permeability, mice were starved overnight, and then FITC-dextran (Sigma-Aldrich, Tokyo, Japan) was administered by oral gavage (44 mg/100 g body weight). After 4 h, mice were anesthetized, blood was collected by cardiac puncture, and mice were sacrificed. Serum was separated from whole blood using BD Microtainer SST Tubes (BD Biosciences), diluted with an equal volume of PBS (pH 7.4), and 100 μL of diluted serum was added to a 96-well microplate. The concentration of FITC in the serum was determined by spectrophotometer fluorometry, with an excitation wavelength of 485 nm and an emission wavelength of 528 nm, using serially diluted FITC-dextran as a standard^35^.

### Method of examining Clostridium butyricum in feces

On the selected days, at least 0.05 g feces were collected and put into a 0.45 ml transport medium. For determination of fecal *C. butyricum* concentrations, *C. butyricum* selective medium was used^36^. Fecal specimens were diluted between 10 × and 100,000 × . Specimens were placed in culture medium separately, smeared evenly, and cultured for 24 h^10^. *C. butyricum* growth was then detected in the culture medium. Viable count was calculated by counting the number of colonies growing on plates, and data converted to equal numbers per gram of feces (CBM-T, CBM + Fidaxo-T). Additionally, we exposed 0.05 mL of undiluted fecal specimens to ethanol^10^. The same process was performed with these samples to reveal the numbers of *C. butyricum* spores in the feces (CBM-AS, CBM + Fidaxo-AS). The testing detectable level is above 2.0 (log amount) per gram of feces.

### Method of examining Clostridioides difficile in feces

Mice were inoculated with *C. difficile* ATCC43255 (10^8–9^ cfu/ml, 75 μl, p.o.) at day 5; treatment was given between days 6 and 10; examination was performed on days 8 and 10. On the selected days, at least 0.05 g feces were collected and put into a 0.45 ml transport medium. For determination of fecal *C. difficile* concentrations, *C. difficile* selective medium (cycloserin cefoxitin mannitol: CCMA agar (Kohjin Bio Co., Ltd., Saitama, Japan)) was used. The fecal specimens were diluted between 10 × and 100,000  × . Specimens were placed in culture medium separately, smeared evenly, and cultured for 48 h. *C. difficile* growth was then detected in the culture medium. Viable count was calculated by counting the number of colonies growing on plates, and data converted to equal numbers in per gram of feces. The testing detectable level is above 2.0 (log amount) per gram of feces.

### DNA extraction

To characterize the microbiome composition in the colon, fecal samples from each mouse were analyzed by sequencing the V3-V4 regions of the 16S rRNA gene^10^. Bacterial DNA extraction from 200 μL of the suspension was performed by using a magLEAD 12gC (Precision System Science, Chiba, Japan), with MagDEA Dx SV (Precision System Science) as a reagent for the automatic nucleic acid extraction. Briefly, fecal pellets were suspended in 100 mM Tris–HCl and 40 mM EDTA buffer (pH 9.0) and centrifuged (20,800 g, 5 min). After the supernatant was removed, fecal pellets were suspended in 10 mM Tris–HCl and 10 mM EDTA buffer (pH 8.0), and the samples were then beaten with zirconia beads (7,000 rpm, 20 s) using a Magnalyser (Roche, Penzberg, Germany). DNA was extracted from the bead-treated suspensions (100 µl) using a magLEAD 12gC (Precision System Science) with MagDEA Dx SV (Precision System Science)^10^.

### Gut microbiome analysis

Meta 16S rRNA gene sequencing PCR was performed using Ex Taq Hot Start (TAKARA Bio, Shiga, Japan) and the Illumina forward primer 50-AATGATACGGCGACCACCGAGATCTACAC (adaptor sequence) + barcode (eight bases) + ACACTCTTTCCCTACACGACGCTCTTCCGATCT (sequence primer) + CCTACGGGNGGCWGCAG-30 (341F) and the Illumina reverse primer 50-CAAGCAGAAGACGGCATACGAGAT (adaptor sequence) + barcode (eight bases) + GTGACTGGAGTTCAGACGTGTGCTCTTCCGATCT (sequence primer) + GACTACHVGGGTATCTAATCC-30 (805R) to the hypervariable V3–V4 region of the 16Sr RNA gene^10^. Amplicons generated from each sample were subsequently purified using SPRI select (Beckman Coulter, Inc., Brea, CA, USA). DNA was quantified using a Quantus Fluorometer and the QuantiFluor dsDNA System (Promega Corporation, Madison, WI, USA). Mixed samples were prepared by pooling approximately equal amounts of amplified DNA and sequenced using MiSeq Reagent Kit V3 (600 cycle) and MiSeq sequencer (Illumina, San Diego, CA, USA), according to the manufacturer’s instructions.

The 16S rRNA sequence data generated by the MiSeq sequencer (Illumina) were processed by the quantitative insights into microbial ecology (QIIME 1.9.1) pipeline^37,38^. Sequences with an average quality value of < 20 were filtered out, and chimeric sequences were removed using USEARCH^39^. Sequences were clustered into operational taxonomic units (OTUs) based on 97% sequence similarity at the species level using UCLUST^38^ against Green genes database 13_8^40^. A representative sequence for each OTU was aligned with PyNAST^37^. Bacterial taxonomy was assigned using UCLUST^38^. Genomic DNA from 20 Strain Even Mix Genomic Material (ATCC^®^ MSA-1002™) was used in the study to evaluate data analysis procedures.

### Alpha (α)-and beta (β)-diversity analysis

Within-community diversity (α-diversity) was calculated using QIIME. An α-rarefaction was generated using a Chao 1 estimator of species richness with 10 sampling repetitions at each sampling depth^41^. An even depth of 22,884 ~ 54,921 sequences per sample was used for calculation of richness and diversity indices. To compare microbial composition between samples, β-diversity was measured by calculating the weighted unifrac distances using QIIME default scripts^42^. PCoA was applied on the resulting distance matrices to generate two-dimensional plots^11^. Each colored point represents a fecal sample obtained from one mouse coloring is according to different treatments (control, fidaxomicin administration group, CBM 588 administration group, and combination group). *p* values were calculated using PERMANOVA. Samples clustered according to treatment status of the mice (*p* < 0.05).

### In vitro* study to reveal the impact of succinate concentration on Clostridioides difficile proliferation*

*C. difficile* strain ATCC43255 isolate, with median minimum inhibitory concentration (MIC) of 0.06 μg/mL (range 0.03–0.12 μg/mL), was cultured anaerobically (6% H_2_, 20% CO_2_, 74% N_2_) with shaking in modified GAM broth (Nissui Pharmaceutical Co., Ltd.) containing succinate 0–0.5 mg/mL and fidaxomicin 0.01 μg/mL. Colony measurements were taken at 24 h and 48 h (n = 5 per group). The testing detectable level was above 2.0 (log amount) per mL of broth; data shown as mean ± SD (n = 5 per group). The broth specimens were diluted between 10 × and 100,000 × , placed in culture medium separately, smeared evenly, and cultured for 48 h. *C. difficile* growth was then detected in the culture medium. *C. difficile* selective medium (cycloserin cefoxitin mannitol: CCMA agar (Kohjin Bio Co., Ltd., Saitama, Japan) was used. Viable counts were calculated by counting the number of colonies that grew on the plates. Additionally, organic acids in the medium from the cultures after 24 h and 48 h from the start of the incubation were detected by high-performance liquid chromatography (Prominence, SHIMADZU, Kyoto, Japan).

### Organic acids

For in vivo study, on day 8, at least 0.3 g feces were collected from each mouse in the four groups. For determination of organic acids, 0.1 g of feces was placed in a 2.0 mL-tube and suspended in 400 µL 1X PBS. For in vitro study, at 24 h and 48 h from the start of the incubation, 1 ml of medium was placed in 2.0 mL-tube and suspended in 400 µL 1X PBS. Samples were vortexed at 3000 rpm for 1 min at room temperature using Vortex Mixer (Heathrow Scientific, IL, USA), kept on ice for 5 min, vortexed at 3000 rpm for 1 min at room temperature, kept on ice for 1 min, and centrifuged at 10,000 × *g* for 5 min at 4 °C. After that, the supernatant was passed through a 0.45 µm filter. Organic acids (acetate, propionate,* n*-butyrate, iso-valerate, succinate, lactate) in feces were measured with high performance liquid chromatography (Prominence, SHIMADZU, Kyoto, Japan) using a post column reaction with a detector (CDD-10A, SHIMADZU, Kyoto, Japan), tandemly-arranged two columns (Shim-pack SCR-102 (H), 300 mm × 8 mm ID, SHIMADZU, Kyoto, Japan), and a guard column (Shim-pack SCR-102 (H), 50 mm × 6 mm ID, SHIMADZU, Kyoto, Japan). The system was used with a mobile phase (5 mM p-toluenesulfonic acid) and a reaction solution (5 mM p-toluenesulfonic acid, 100 µM EDTA, and 20 mM Bis–Tris). The flow rate and oven temperature were 0.8 mL/min and 40 °C, respectively. Deionized water and 5 mM p-toluenesulfonic acid were used (KANTO Chemical, Tokyo, Japan). The reaction solution was adjusted to use 5 mM p-toluenesulfonic acid, Bis (2-hydroxyethyl) aminotris (hydroxymethyl) methane (Tokyo Chemical Industry, Tokyo, Japan) and ethylenediaminetetraacetic acid (KANTO Chemical, Tokyo, Japan). For in vitro study, at 48 h, 1.0 ml samples were collected from all groups. For detection of organic acids, sample preparations were same with fecal samples^10^.

The detector cell temperature was kept at 48 °C. The sample in a 1.0 mL disposable vial (228–31,600-91, SHIMADZU, Kyoto, Japan) was kept at 4 ˚C in a sample cooler (SIL-20AC, SHIMADZU, Kyoto, Japan), and injected 10 µL at a time using an auto-injector. Detection limits for acetic acid, propionic acid, butyric acid, iso-butyric acid, succinic acid, lactic acid, formic acid, valeric acid, and iso-valeric acid were 0.05, 0.05, 0.1, 0.1, 0.05, 0.05, 0.1, 0.1, and 0.1 mM, respectively. Succinic acid (KANTO Chemical), lithium DL-lactate (FUJIFILM Wako Pure Chemical), sodium formate (KANTO Chemical), sodium acetate trihydrate (KANTO Chemical, Tokyo, Japan), sodium propionate (KANTO Chemical), sodium isobutyrate (KANTO Chemical), n-sodium butyrate (KANTO Chemical), and sodium isovalerate (KANTO Chemical) were dissolved in deionized water, and adjusted for a mixed preparation. The concentration of succinic acid, lithium DL-lactate, sodium formate, sodium acetate trihydrate, sodium propionate, sodium isobutyrate, n-sodium butyrate, and sodium isovalerate were 25, 50, 50, 50, 50, 50, 50, and 50 mM respectively, and diluted to use to measure the calibration curve^10^.

The quantification analysis was performed by LabSolutions version5.90 (SHIMADZU) using the peak area and retention time. The quantitative value used the peak area. The concentrations to measure the calibration curve for acetic acid, propionic acid, butyric acid, iso-butyric acid, lactic acid, formic acid, valeric acid, and iso-valeric acid were 0.4, 0.8, 1.2, 1.6, and 2.0 mM, and the concentrations to measure the calibration curve for succinic acid were 0.2, 0.4, 0.6, 0.8, 2.0 mM^10^.

### RNA isolation and preparation of cDNA

To analyze the RNA expression of intracellular cytokines, total RNA was isolated by homogenizing mouse colons (30 mg) using the RNA isolation Kit (MACHEREY–NAGEL, Düren, Germany) in accordance with manufacturer’s instructions. For preparation of complementary DNA (cDNA), Highcapacity RNA to cDNA kit (Thermofisher, Waltham, MA, USA) was used in accordance with manufacturer’s instructions. Total RNA solution (9 μL) was added to 2 × RT Buffer mix and 20 × RT Enzyme Mix for a final volume of 20 μL. The mixture was incubated at 37 °C for 60 min, and stopped by heating to 95 °C for 5 min and holding at 4 °C. For convenience, the incubation was performed in a thermal cycler. The cDNA was either immediately used in real-time PCR applications or placed for long-term storage in a freezer (-20 °C)^11^.

### Quantitative real-time PCR

Quantitative RT-PCR analysis was performed using the PowerUp™ SYBR™ Green PCR Master Mix (Applied Biosystems, Waltham, MA, USA) in accordance with the manufacturer’s protocol^11^. Briefly, PCR reactions were performed in a reaction mixture (10 μL) containing 2 μL of cDNA, 5 μL of PowerUp™ SYBR™ Green PCR Master Mix, 0.5 μL of 10 μM forward primer, 0.5 μL of 10 μM reverse primer, and 2 μL of nuclease-free water. The sequences of primers are summarized in Table S1. Amplification conditions were 2 min at 50 °C, 2 min at 95 °C, and 40 cycles of denaturation at 95 °C for 15 s with annealing/extension at 60 °C for 1 min, when Tm ≥ 60 °C. Amplification conditions were 2 min at 50 °C, 2 min at 95 °C, with 40 cycles of denaturation at 95 °C for 15 s, annealing at 60 °C for 15 s min, and extension at 72 °C for 1 min, when Tm < 60 °C. For relative quantitation, we compared the amount of target normalized to an endogenous reference, β-actin. The formula 2ΔΔCt represents the n-fold differential expression of a specific gene in a treated sample compared with the control, where Ct is the mean of threshold cycle, ΔCt is the difference in Ct values for the target gene and the reference gene, β-actin (in each sample), and ΔΔCt represents the difference between the Ct from the control and each datum. We validated this method by first comparing the standard curves of the reference and the target to show that the efficiencies were equal. Immediately after amplification, the melting curve protocol was utilized to guarantee minimization in primer dimers and other non-specific products. Expression of target genes was analyzed by the ΔΔCt method. Relative RNA expression levels of each target gene were normalized to the control group (represented as RQ)^11^.

### Cytokine studies

The colon tissue samples were homogenized in a RIPA buffer supplemented with protease inhibitors (Nacalai tesque, Kyoto, Japan)^11^. After sonication for 10 s, the suspension was centrifuged at 10,000 × g for 20 min at 4 °C. Protein expression of IL-6, IL-4, IL-10, IL-17, transforming growth factor-β_1_ (TGF-β_1_), and tumor necrosis factor-α (TNF-α) in the supernatants were measured by commercially available mouse cytokine ELISA kits (BioLegend CNS, Inc., San Diego, CA, USA). Protein expression of immunoglobulin A (IgA) in the supernatants were measured by commercially available mouse cytokine ELISA kits (Invitrogen, Carlsbad, CA, USA). Protein expression of Mucine (MUC)-2 and Zonula occludens protein 1 (ZO-1) were also measured by commercially available mouse ELISA kits (Cusabio technology, Houston, TX, USA and LSBio, Seattle, WA, USA). These procedures were performed according to the manufacturer’s instructions.

### Isolation of lymphocytes from colon tissue

To isolate colon lumina propria lymphocytes, large intestines were collected and opened longitudinally, washed to remove fecal content, and shaken in HBSS containing 1.5% fetal bovine serum (FBS) and 0.5 M EDTA for 20 min at 37 °C. After removing epithelial cells and fat tissue, the intestines were cut into small pieces and incubated with HBSS containing 1.5% FBS and 1 mg/ml collagenase type IV (Worthington Biochemical Corporation, Lakewood, NJ, USA) for 1 h at 37 °C in a shaking water bath. The digested tissues were processed through a 100 μm filter (pluriSelect, Germany) and resuspended in 5 ml of 40% Percoll (GE Healthcare, Chicago, IL, USA) before overlay on 5 ml of 80% Percoll in a 15-ml Falcon tube. Percoll gradient separation was performed by centrifugation at 800 g for 20 min at 20 °C. The interface cells were collected and used as LP lymphocytes^11^.

### Stimulation of LPMCs

Lumina propria mononuclear cells (LPMCs) were seeded on 96-well tissue culture plates (1 × 10^6^ cells/ml) and incubated for 24 h at 37 °C in a humidified incubator with 5% CO_2_. For measurement of secreted cytokines, culture supernatants were collected and stored at − 80 °C^11^.

### Flow cytometry analysis

Collected cells were resuspended in RPMI 1640 containing 10% FBS. For detection of IL-17A, lymphocytes were stimulated for 4 h with 50 ng/mL PMA (Sigma) and 1 μg/mL ionomycin (Sigma). Cells were first stained for surface CD3 and CD4, then fixed and permeabilized using a BD Cytofix/Cytoperm kit (554,714, BD Biosciences), and finally stained for intracellular IL-17A^11^. For detection of plasma B cell, lymphocytes were stimulated for 4 h with 50 ng/mL PMA (Sigma-Aldrich) and 1 μg/mL ionomycin (Sigma-Aldrich). Cells were first stained for surface CD45R/B220, CD19, and CD138. The following antibodies were used: FITC-labeled anti-CD4 cell (561,535, BD Biosciences), Per-CP-labeled anti-CD3 Ab (561,089, BD Biosciences), PE-labeled anti-CD8 Ab (561,095, BD Biosciences), FITC-labeled anti-CD45R/B220 Ab (553,087, BD Biosciences), APC-labeled anti-CD19 Ab (550,992, BD Biosciences), Brilliant violet 421-labeled anti-mouse CD138 Ab (142,507, Syndecan-1), and PE-labeled anti-IL-17A Ab (559,502, BD Biosciences). Flow cytometry was performed using FACSCant™ II (BD Biosciences) and data were analyzed with FlowJo software (TreeStar Inc, Williamson Way Ashland, OR, USA)^11^.

### Statistics and analysis

For *C. butyricum* colony counts, all the data were presented as the mean ± SD. Unpaired *t*-tests were performed using JMP. *p* < 0.05 was the level of significance^10^. For microbiome analysis, all data in bar graph or dot plot formats are expressed as mean ± SD. Differences were considered statistically significant at *p* < 0.05^10^. For the microbiome data, Kruskal–Wallis analysis (non-parametric equivalent to ANOVA) was used to compare average proportions of each taxon level (phylum, class, order, family, and genus) in mouse fecal samples. Mann–Whitney U test was used to determine significant differences between each treatment group^10^. Other statistical analysis of the quantitative multiple group comparisons was performed using a one-way analysis of variance followed by Tukey’s test, *p < 0.05. Statistical analysis of the quantitative two group comparisons was performed using a Student t-test, *p < 0.05^10,11^.

## Supplementary Information

Supplementary Information 1.

Supplementary Information 2.

## Abbreviations

APRIL: Proliferation-inducing ligand
BAFF: B-cell activating factor
CBM 588: *Clostridium butyricum* MIYAIRI 588
CDI: *Clostridioides difficile* Infection
CLDN4: Claudins-4
cLP: Colon lumina propria
CSR: Class-switch recombination
DCs: Dendritic cells
IgA: Immunoglobrin A
IBD: Inflammatory bowel disease
IL: Interleukin
MIC: Minimum inhibitory concentration
MUC-2: Mucin 2
OCLD: Occluding
PCoA: Principal coordinate analysis
pIgR: Epithelial polymeric immunoglobulin receptor
PPs: Peyer's patchs
SUCNR1: Succinate receptor 1
Tfh cell: T follicular helper cell
TGF-β1: Transforming growth factor-β1
Th17 cell: T helper 17 cell
TJ: Tight junction
TNF-α: Tumor necrosis factor-α
UC: Ulcerative colitis
ZO-1: Zonula occludens-1
