# Supplementary material for: Clostridium butyricum enhances colonization resistance against Clostridioides difficile by metabolic and immune modulation
Source: Sci Rep. 2021 Jul 22;11:15007. doi: 10.1038/s41598-021-94572-z (PMC8298451; doi:10.1038/s41598-021-94572-z)
Supplement: Supplementary file 1 — Supplementary Information 1. [file 41598_2021_94572_MOESM1_ESM.docx]

**Title: *Clostridium butyricum* enhances colonization resistance against *Clostridioides difficile* by metabolic and immune modulation**

Mao Hagihara, Tadashi Ariyoshi, Yasutoshi Kuroki, Shuhei Eguchi, Seiya Higashi, Takeshi Mori, Tsunemasa Nonogaki, Kenta Iwasaki, Makoto Yamashita, Nobuhiro Asai, Yusuke Koizumi, Kentaro Oka, Motomichi Takahashi, Yuka Yamagishi, Hiroshige Mikamo

**Supporting Information**

**Table S1.** Primers used for quantitative real-time RT-PCR, Related to Figure 5C and 7B

| Name |  | Sequence | Reference |
| --- | --- | --- | --- |
| β-Actin | Forward (5’→3’) | GTG CCG CCT GGA GAA ACC | Zheng et al., 2017 |
|  | Reverse (5’→3’) | GGT GGA AGA GTG GGA GTT GC |  |
| APRIL | Forward (5’→3’) | AGA GGT GAT GTG GCA ACC AGT A | Qin et al., 2018 |
|  | Reverse (5’→3’) | CCC TTG TCC TTC CCG AGA TA |  |
| BAFF | Forward (5’→3’) | GGT GAC CCT GTT CCG ATG TAT T | Qin et al., 2018 |
|  | Reverse (5’→3’) | GAA GGT GTC GTC TCC GTT GC |  |
| MUC-2 | Forward (5’→3’) | TTT CAA GCA CCC CTG TAA CC | Song et al., 2018 |
|  | Reverse (5’→3’) | AGG TCC TGG TGT TGA ACC TG |  |
| ZO-1 | Forward (5’→3’) | ACT ATG ACC ATC GCC TAC GG | Song et al., 2018 |
|  | Reverse (5’→3’) | GGG GAT GCT GAT TCT CAA AA |  |
| OCLN | Forward (5’→3’) | CGG TAC AGC AGC AAT GGT AA | Song et al., 2018 |
|  | Reverse (5’→3’) | CTC CCC ACC TGT CGT GTA GT |  |
| CLDN4 | Forward (5’→3’) | GGG GAT CAT CCT GAG TTG TG | Song et al., 2018 |
|  | Reverse (5’→3’) | CAC TGC ATC TGA CCT GTG CT |  |

APRIL: a proliferation-inducing ligand, BAFF: B cell activating factor belonging to the tumor necro- sis factor family, MUC-2: Secretory protein mucin-2, ZO-1: Zonula occludens-1, OCLD: occludin, CLDN4: claudins-4.

**Reference**

1. Qin, W., Wang, L., Zhai, R., Ma, Q., Liu, J., Bao, C., Sun, D., Zhang, H., Sun, C., Feng, X., et al. Apa2H1, the first head domain of Apa2 trimeric autotransporter adhesin, activates mouse bone marrow-derived dendritic cells and immunization with Apa2H1 protects against Actinobacillus pleuropneumoniae infection. Mol. Immunol. 81, 108-117 (2017).
2. Song, C.H., Kim, N., Sohn, S.H., Lee, S.M., Nam, R.H., Na, H.Y., Lee, D.H., and Surh, Y.J. Effects of 17β-Estradiol on Colonic Permeability and Inflammation in an Azoxymethane/Dextran Sulfate Sodium-Induced Colitis Mouse Model. Gut Liver 12, 682-693 (2018).
3. Zheng, L., Zhang, Y.L., Dai, Y.C., Chen, X., Chen, D.L., Dai, Y.T., and Tang, Z.P. Jianpi Qingchang decoction alleviates ulcerative colitis by inhibiting nuclear factor-κB activation. World J. Gastroenterol. 23, 1180-1188 (2017).
