# Supplementary material for: Clostridium butyricum enhances colonization resistance against Clostridioides difficile by metabolic and immune modulation
Source: Sci Rep. 2021 Jul 22;11:15007. doi: 10.1038/s41598-021-94572-z (PMC8298451; doi:10.1038/s41598-021-94572-z)
Supplement: Supplementary file 2 — Supplementary Information 2. [file 41598_2021_94572_MOESM2_ESM.pptx]

## Slide 1
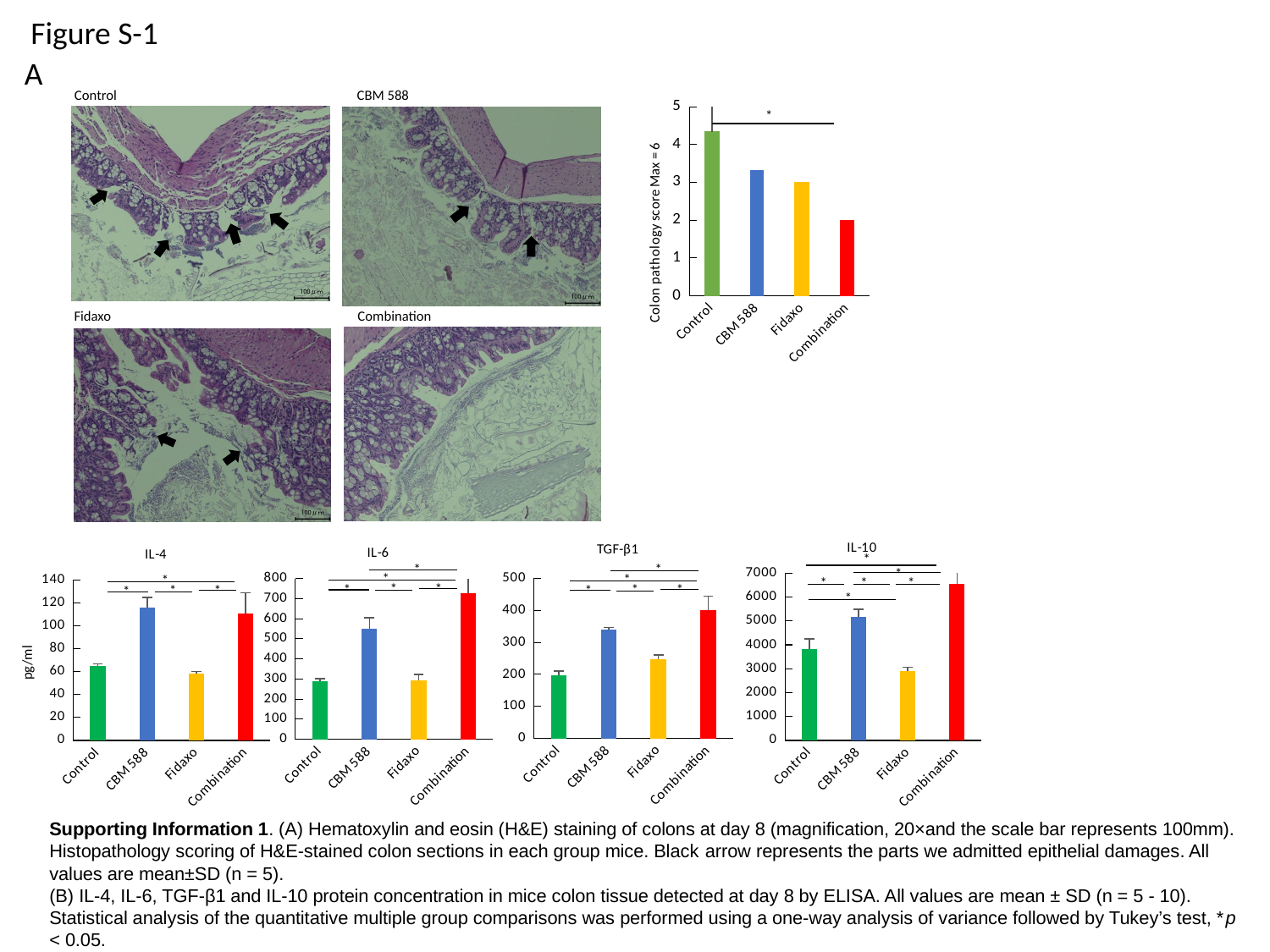

Figure S-1
A
Control
CBM 588
### Chart
| Category | |
|---|---|
| Control | 4.333333333333333 |
| CBM 588 | 3.3333333333333335 |
| Fidaxo | 3.0 |
| Combination | 2.0 |*
Fidaxo
Combination
### Chart: IL-10
| Category | |
|---|---|
| Control | 3820.8 |
| CBM 588 | 5154.4 |
| Fidaxo | 2884.0 |
| Combination | 6533.6 |
### Chart: IL-6
| Category | |
|---|---|
| Control | 288.65384615384613 |
| CBM 588 | 551.3461538461539 |
| Fidaxo | 294.80769230769226 |
| Combination | 725.5769230769231 |
### Chart: TGF-β1
| Category | Average |
|---|---|
| Control | 197.25000000000006 |
| CBM 588 | 341.75 |
| Fidaxo | 248.41666666666666 |
| Combination | 401.5833333333333 |
### Chart: IL-4
| Category | |
|---|---|
| Control | 64.59677419354838 |
| CBM 588 | 115.88709677419357 |
| Fidaxo | 58.225806451612904 |
| Combination | 110.3225806451613 |*
*
*
*
*
*
*
*
*
*
*
*
*
*
*
*
*
*
*
*
pg/ml
Supporting Information 1. (A) Hematoxylin and eosin (H&E) staining of colons at day 8 (magnification, 20×and the scale bar represents 100mm). Histopathology scoring of H&E-stained colon sections in each group mice. Black arrow represents the parts we admitted epithelial damages. All values are mean±SD (n = 5).
(B) IL-4, IL-6, TGF-β1 and IL-10 protein concentration in mice colon tissue detected at day 8 by ELISA. All values are mean ± SD (n = 5 - 10). Statistical analysis of the quantitative multiple group comparisons was performed using a one-way analysis of variance followed by Tukey’s test, *p < 0.05.

## Slide 2
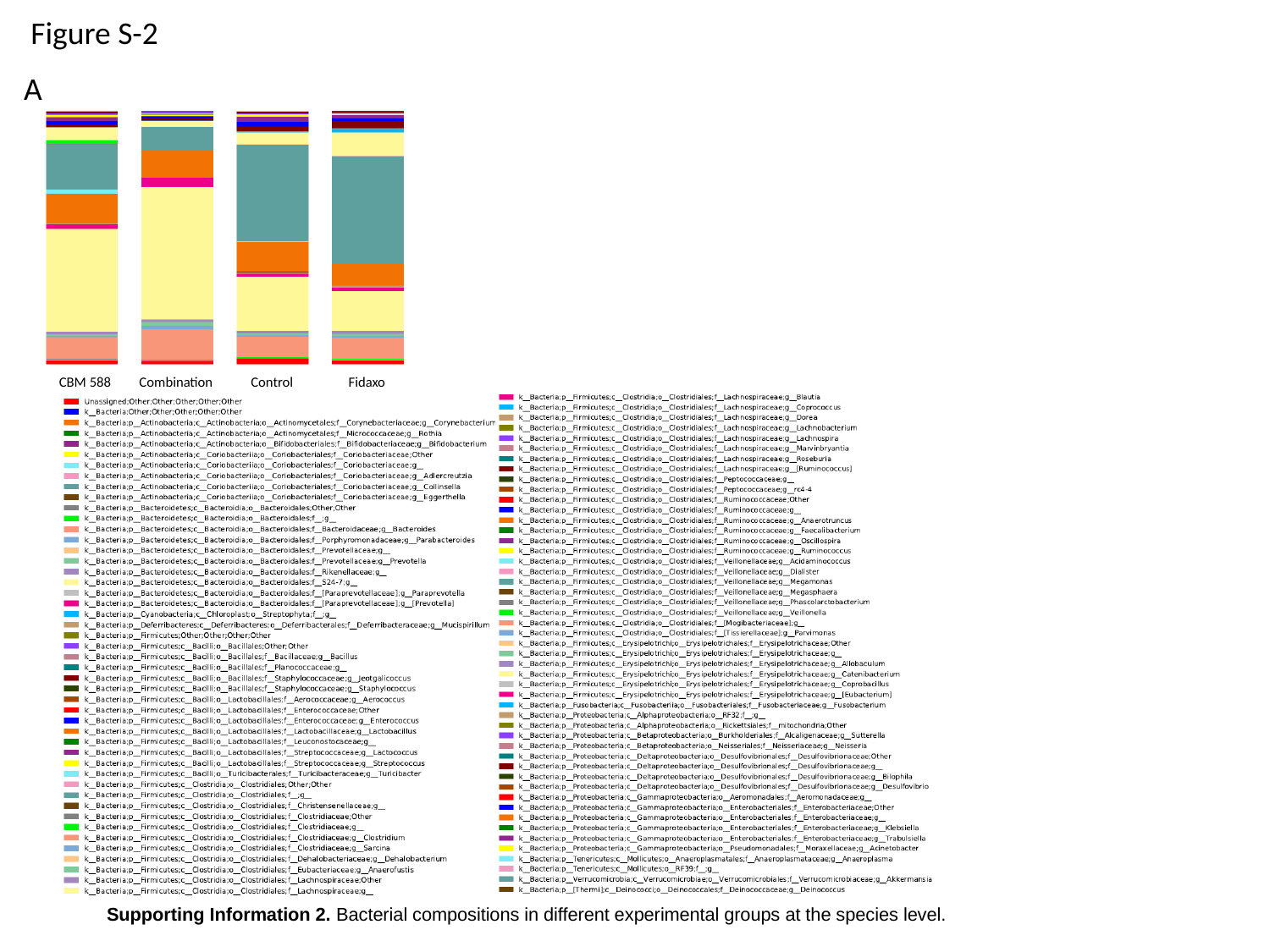

Figure S-2
A
CBM 588
Combination
Control
Fidaxo
Supporting Information 2. Bacterial compositions in different experimental groups at the species level.

## Slide 3
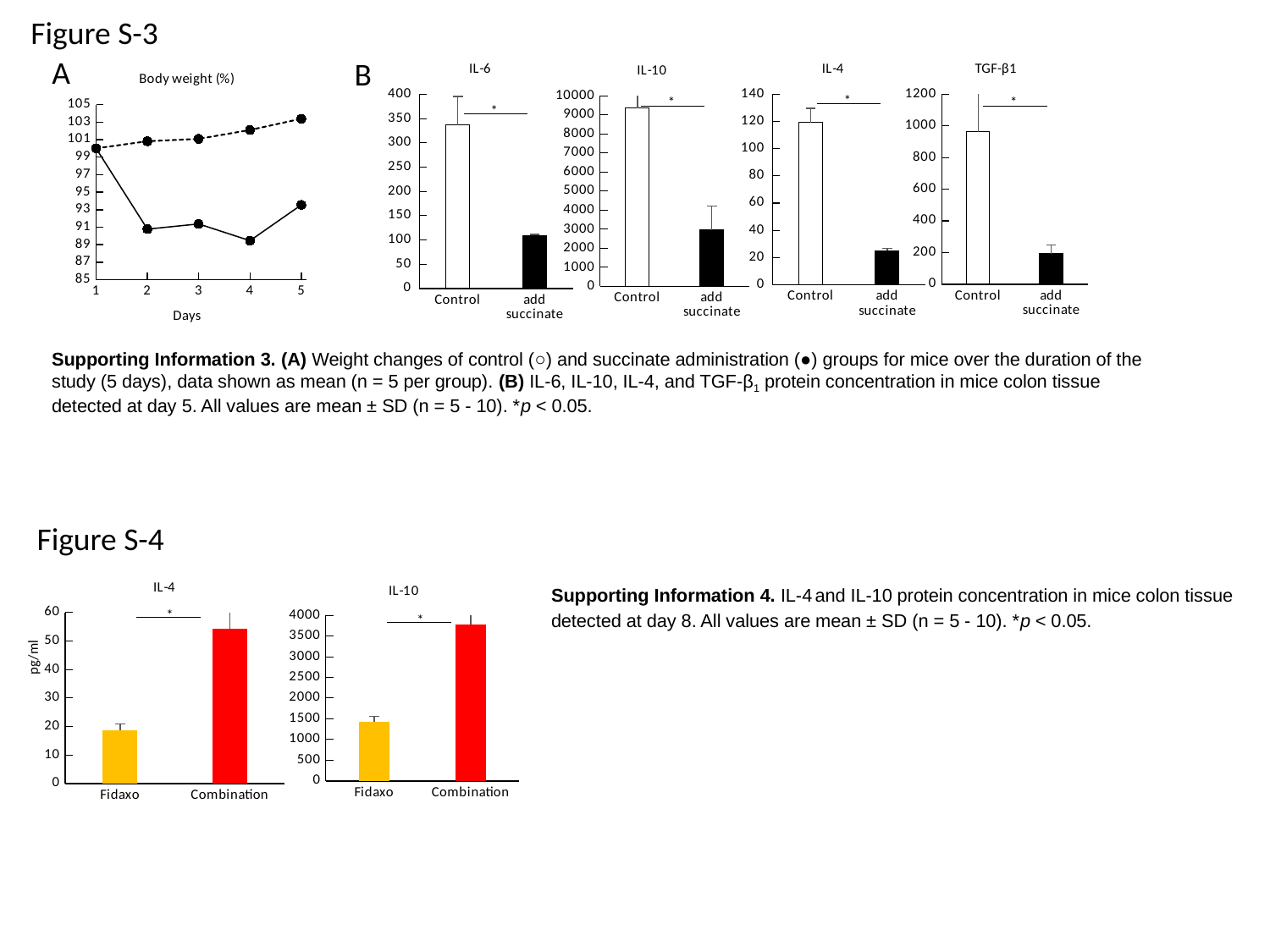

Figure S-3
A
### Chart: IL-6
| Category | |
|---|---|
| Control | 336.50641025641033 |
| add succinate | 109.26282051282051 |
### Chart: IL-4
| Category | |
|---|---|
| Control | 119.31451612903226 |
| add succinate | 25.0268817204301 |
### Chart: TGF-β1
| Category | |
|---|---|
| Control | 962.5 |
| add succinate | 193.83333333333334 |B
### Chart: IL-10
| Category | |
|---|---|
| Control | 9351.6 |
| add succinate | 2954.8 |
### Chart: Body weight (%)
| Category | Control | add Succinate |
|---|---|---|*
*
*
*
Supporting Information 3. (A) Weight changes of control (○) and succinate administration (●) groups for mice over the duration of the study (5 days), data shown as mean (n = 5 per group). (B) IL-6, IL-10, IL-4, and TGF-β1 protein concentration in mice colon tissue detected at day 5. All values are mean ± SD (n = 5 - 10). *p < 0.05.
Figure S-4
### Chart: IL-4
| Category | |
|---|---|
| Fidaxo | 18.70967741935484 |
| Combination | 54.395161290322584 |
### Chart: IL-10
| Category | |
|---|---|
| Fidaxo | 1417.0 |
| Combination | 3784.6 |Supporting Information 4. IL-4 and IL-10 protein concentration in mice colon tissue detected at day 8. All values are mean ± SD (n = 5 - 10). *p < 0.05.
*
*
pg/ml

## Slide 4
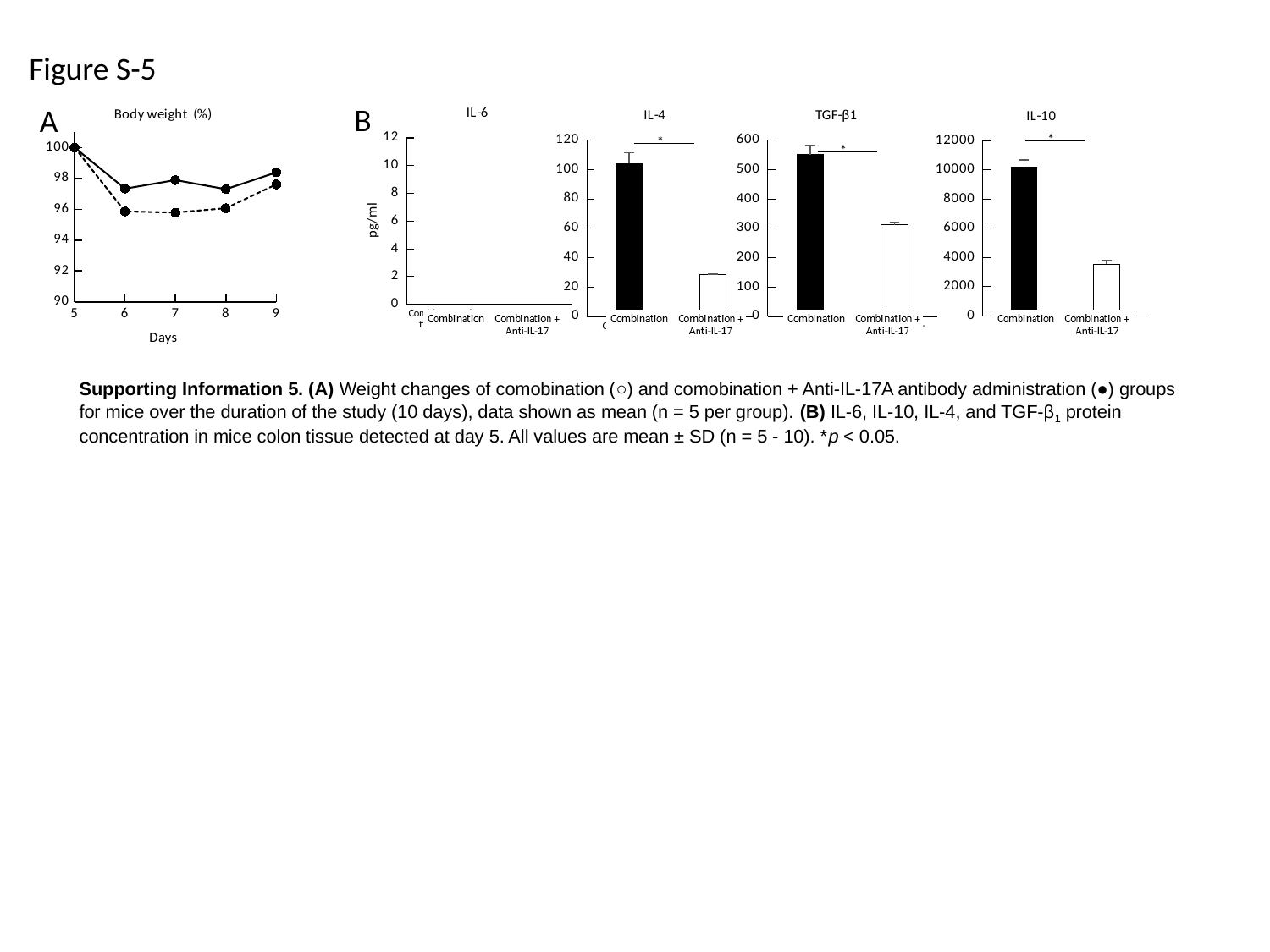

Figure S-5
### Chart: Body weight (%)
| Category | Fidaxo+CBM 588 | Fidaxo+CBM 588+Anti-IL-17 antibody |
|---|---|---|
### Chart: IL-6
| Category | |
|---|---|
| Combination | 624.2948717948719 |
| Anti-IL-17 | 590.9615384615386 |
### Chart: IL-4
| Category | |
|---|---|
| Combination | 104.19354838709678 |
| Combination + Anti-IL-17 | 28.454301075268816 |
### Chart: TGF-β1
| Category | |
|---|---|
| Combination | 550.4722222222223 |
| Combination + Anti-IL-17 | 311.5833333333333 |B
A
### Chart: IL-10
| Category | |
|---|---|
| Combination | 10203.333333333332 |
| Anti-IL-17 | 3547.3333333333335 |*
*
*
pg/ml
Supporting Information 5. (A) Weight changes of comobination (○) and comobination + Anti-IL-17A antibody administration (●) groups for mice over the duration of the study (10 days), data shown as mean (n = 5 per group). (B) IL-6, IL-10, IL-4, and TGF-β1 protein concentration in mice colon tissue detected at day 5. All values are mean ± SD (n = 5 - 10). *p < 0.05.
